# Integrated metabolome and transcriptome analysis provide insight into the biosynthesis of flavonoids in *Panax japonicus*

**DOI:** 10.3389/fpls.2024.1432563

**Published:** 2024-07-29

**Authors:** ChunYu Chen, Ping Wang, Yan Yan, ZeWei Jiao, ShuHao Xie, Ye Li, Peng Di

**Affiliations:** ^1^ Chongqing Three Gorges Medical College, Chongqing, China; ^2^ Chongqing Key Laboratory of Development and Utilization of Genuine Medicinal Materials in Three Gorges Reservoir Area, Chongqing, China; ^3^ State Local Joint Engineering Research Center of Ginseng Breeding and Application, College of Chinese Medicinal Materials, Jilin Agricultural University, Changchun, China

**Keywords:** *Panax japonicus*, metabolome, transcriptome, flavonoids, biosynthesis pathway

## Abstract

*Panax japonicus* is an important medicinal plant, and flavonoids are one of its main secondary metabolites. In this study, the main roots, fibrous roots, stems, leaves and flowers of *P. japonicus* were analyzed using transcriptomics and widely targeted metabolomics. Through correlation analysis of transcription and metabolism, the flavonoid biosynthesis pathway in *P. japonicus* was analyzed, and the accumulation of flavonoid metabolites and the expression of related genes were investigated. Metabolomics revealed a total of 209 flavonoid metabolites in *P. japonicus*, among which flavonoids, flavonols, flavanones and flavanonols significantly accumulated in the flowers and leaves. Transcriptome sequencing revealed that key genes in the flavonoid pathway exhibited increased expression in the flowers and leaves. The expression patterns of key genes involved in flavonoid biosynthesis, including *PjC4H*, *Pj4CL*, *PjCHS*, *PjCHI*, *PjF3H*, *PjF3’H*, *PjCYP*, and *PjPAL*, are consistent with their upstream and downstream metabolites, demonstrating a significant positive correlation among them. In addition, the *PjUGT* gene is highly expressed in five tissues of *P. japonicus*, indicating that PjUGT is one of the key factors for the diversity of flavonoid glycosides. The WGCNA results showed that WRKY transcription factors exist widely in the candidate modules, and it was possible that *PjWRKY* transcription factors are involved in regulating the expression of key genes involved in flavonoid biosynthesis and the biosynthesis of flavonoid metabolites. This study reveals spatial differences in the accumulation patterns of flavonoid metabolites in different tissues and provides important clues for further understanding the regulatory mechanisms of flavonoid metabolism in *P. japonicus*, thus contributing to the optimization of germplasm resources of *P. japonicus* and the promotion of genetic diversity analysis.

## 1 Introduction

*Panax japonicus* is a plant belonging to the *Panax genus* in the *Araliaceae* family and is primarily found in China, Japan, and South Korea (Yang et al., 2014; Zhang et al., 2015; Morita et al., 1985). *P. japonicus* has similar components to *Panax ginseng* and *Panax notoginseng* and is used for the medicinal functions of nourishing and strengthening, eliminating stasis to stop pains, as well as suppressing cough and dispelling phlegm (Ngan et al., 1999). Flavonoids are among the key metabolites of *P. japonicus*, and it is therefore worthwhile to explore the flavonoid biosynthesis of secondary metabolites. Currently, scholars worldwide have investigated the extraction and content of total flavonoids in natural medicinal plants. In plants, natural flavonoids are internal signal molecules, intermediates or metabolites of polyphenols (Roleira et al., 2018), and their main types include flavones, isoflavones, flavanols, flavanones, flavanonols, chalcones, anthocyanidins and flavanols (Ververidis et al., 2007).

Flavonoids not only have pharmacological activities but can also enhance the adaptability of plants to various terrestrial environments and actively participate in the ecological defense mechanism of plants (Dixion, 2001; Chen and Chen, 2013). To date, more than 8,000 flavonoids have been detected in plants, and flavonoids are distributed in roots, stems, leaves, flowers, fruits and other plant tissues (Wen et al., 2020). Specifically, flavonoids can promote the growth of plants (Stafford, 1991), enhance the resistance of plants to diseases (Agati et al., 2012), effectively reduce the damage caused by ultraviolet radiation to plants (Harbone et al., 2000), scavenge oxygen free radicals (Agati et al., 2009), drive away foragers and prevent the invasion of harmful microorganisms (Falcone Ferreyra et al., 2012). In previous studies, the mechanism of flavonoid biosynthesis in *Arabidopsis thaliana*, rice and tomato plants has been analyzed (Deng and Lu, 2017; Ma and Constabel, 2019; Yonekura-Sakakibara et al., 2019). For medicinal plants, research on the molecular regulation of flavonoids has gradually increased. The SbMYB2 and SbMYB7 genes in *Scutellaria baicalensis* have been shown to increase the accumulation of flavonoids in transgenic tobacco (Qi et al., 2015). After MeJA treatment of *Salvia miltiorrhiza*, the SmERF115 transcription factor has been shown to upregulate the biosynthesis of phenolic acids (Sun et al., 2019). In addition, the ubiquitination-mediated degradation of PhCHS by the PhRING-H2 protein is an important posttranslational regulatory mechanism that regulates the biosynthesis of flavonoids in *Paeonia lactiflora* (Gu et al., 2019).

Transcriptomics is a powerful tool for studying functional genes (Parkhomchuk et al., 2009), and the differentially expressed genes (DEGs) and regulatory network information obtained through transcriptome sequencing help reveal the roles and regulatory mechanisms of functional genes in biological processes (Covington et al., 2008). Metabolomics is widely used in the fields of gene function analysis, metabolic pathway research, and metabolic regulatory mechanism research to increase yield and improve quality. It is estimated that the total number of plant metabolites ranges from 200,000 to 1,000,000. *A. thaliana* alone is predicted to contain more than 5,000 primary and secondary metabolites (Dixon and Strack, 2003). The biosynthesis pathways of plant secondary metabolites are diverse and complex, and traditional research methods cannot meet the needs of research on medicinal plant secondary metabolites. Multiomics technology has been widely applied in the study of secondary metabolism in medicinal plants (Wang et al., 2017). For example, a combined transcriptomic analysis of the biosynthetic pathway of tanshinone in *S. miltiorrhiza* has been previously performed (Gao et al., 2014). In *Schisandra chinensis* research, the candidate genes involved in the biosynthesis of lignans in fruit were determined by transcriptomics and metabonomics analysis (Sun et al., 2023). A comprehensive network of flavonoid biosynthesis and regulation was obtained through transcriptomics and targeted metabolomics analysis of the rhizome of *Fagopyrum cymosum* (Huang et al., 2022). An integration of metabolic and transcriptional analyses revealed that DEGs of the chalcone synthase (CHS) gene are a major factor in the variations in flavonoid species and content among differently colored *Carthamus tinctorius* (safflower). A significant correlation was also revealed between the uridine diphosphate glucose glycosyltransferase gene CtUGT9 and volatile carbohydrates in safflower (Ren et al., 2022). In *Dendrobium* studies, the optimal harvesting period for three Dendrobium species was determined, and key genes involved in flavonoid biosynthesis were identified (Yuan et al., 2022).

To date, research on flavonoids in *P. japonicus* has focused mainly on the extraction, separation and preparation of their chemical components. For example, the extraction and determination methods of total flavonoid glycosides from *P. japonicus* in Guizhou were explored in detail, and the alkaline extraction process of total flavonoids from the stems and leaves of *P. japonicus* was optimized by orthogonal experiments (Zhang et al., 2009). However, the distribution of flavonoids in different tissues of *P. japonicus* and the related genes influencing the variation in flavonoid accumulation have not yet been reported. In this study, the changes in flavonoid biosynthesis in different tissue parts (main roots, fibrous roots, stems, leaves and flowers) of *P. japonicus* were investigated from genetic and chemical perspectives by combining broad target metabolomics and transcriptomics. A total of seven categories (209 flavonoids) were detected, and the expression profiles of flavonoid biosynthesis-related genes in *P. japonicus* were obtained. In this study, the molecular mechanism of flavonoid changes in *P. japonicus* was further explored at the molecular level, and the dynamic changes and biosynthetic pathways of flavonoids in *P. japonicus* were revealed, thereby providing a valuable basis and reference for breeding and flavonoid biosynthesis in *P. japonicus*.

## 2 Materials and methods

### 2.1 Plant material preparation and extraction for widely targeted metabolic profiling

Four-year-old *P. japonicus* cultivated artificially in Yunwu Township, Fengjie County, Chongqing. The main roots, fibrous roots, stems, leaves, and flowers of *P. japonicus* were collected, and three sets of biological replicates were performed for each tissue: mRM1, mRM2, mRM3, fRM1, fRM2, fRM3, SM1, SM2, SM3, LM1, LM2, LM3, FM1, FM2, and FM3. All the samples were immediately frozen in liquid nitrogen and then stored at −80°C for subsequent extraction of RNA and metabolites.

Initially, a vacuum freeze-dryer (Model Scientz-100F) was utilized to freeze-dry the biological samples. Subsequently, the freeze-dried sample was pulverized using a mixer mill (MM 400, Retsch) equipped with zirconia beads for 1.5 min at a frequency of 30 Hz. For extraction, 50 mg of the lyophilized powder was dissolved in 1.2 mL of a 70% methanol solution. The mixture was vortexed for 30 sec every 30 min, and the process was repeated a total of six times. Following centrifugation at 12,000 rpm for 3 min, the extracts were filtered through a filter with a pore size of 0.22 µm (SCAA-104, ANPEL, Shanghai, China; http://www.anpel.com.cn/) before undergoing UPLC-MS/MS analysis.

### 2.2 UPLC-MS/MS conditions

The sample extracts were analyzed using a UPLC-ESI-MS/MS system (UPLC, ExionLC™ AD, https://sciex.com.cn/; MS, Applied Biosystems 4500 Q TRAP, https://sciex.com.cn/). The analytical conditions were as follows: UPLC: column, Agilent SB-C18 (1.8 µm, 2.1 mm * 100 mm). The mobile phase consisted of solvent A, pure water with 0.1% formic acid; solvent B, acetonitrile with 0.1% formic acid. Sample measurements were performed with a gradient program that employed the starting conditions of 95% A, 5% B. Within 9 min, a linear gradient to 5% A, 95% B was programmed, and a composition of 5% A, 95% B was maintained for 1 min. Subsequently, a composition of 95% A, 5% B was adjusted within 1.1 min and maintained for 2.9 min. The flow velocity was set to 0.35 mL per minute; the column oven was set to 40°C; and the injection volume was 4 μL. The effluent was alternatively connected to an ESI-triple quadrupole-linear ion trap (QTRAP)-MS instrument.

The ESI source operation parameters were as follows: source temperature, 550°C; ion spray voltage (IS), 5500 V (positive ion mode)/-4500 V (negative ion mode); ion source gas I (GSI); gas II (GSII); and curtain gas (CUR) were set at 50, 60, and 25 psi, respectively; and collision-activated dissociation (CAD) was high. QQQ scans were acquired as MRM experiments with the collision gas (nitrogen) set to medium. DP (declustering potential) and CE (collision energy) for individual MRM transitions were performed with further DP and CE optimization. A specific set of MRM transitions was monitored for each period according to the metabolites eluted within this period.

MetaboAnalyst 6.0 (https://www.metaboanalyst.ca/faces/home.xhtml) was used to analyze the differentially abundant metabolites, and fold change (FC) ≥ 2 or < 0.5 and P < 0.05 were set as the thresholds for the differentially abundant metabolites. A volcano plot was drawn online. PCA was performed at https://www.bioinformatics.com.cn, an online platform for data analysis and visualization. A Venn diagram was drawn via the Even online website (http://www.ehbio.com/test/Venn/#/) (this method is used for subsequent Venn diagram drawing). The metabolite heatmap was drawn using TBtools-ll v2.084.

### 2.3 Transcriptome analysis

Total RNA from the main roots, fibrous roots, stems, leaves and flowers of *P. japonicus* was extracted by using a plant RNA extraction kit (Beijing TransGen Biotechnology Co., Ltd.), and the resulting strains were named mRT1, mRT2, mRT3, fRT1, fRT2, fRT3, ST1, ST2, ST3, LT1, LT2, LT3, FT1, FT3 and FT5. The concentration and purity of the RNA samples were detected by a NanoPhotometer N50 (Implen, Germany). RNA with good purity and integrity was selected for reverse transcription. Poly(A)RNA was purified from total RNA (5 μg) by two rounds of purification using magnetic beads attached to poly(T) oligonucleotides. After purification, the mRNA was segmented into divalent cation fragments at high temperatures. Then, according to the instructions of the mRNASeq sample preparation kit (Illumina, USA), the cleaved RNA fragments were reverse transcribed to create the final cDNA library, and the average insert size of the paired-end library was 300 bp (± 50 bp). After the library passed the quality inspection, 5’ and 3’ sequences were sequenced by Nuohe Zhiyuan using the Illumina HiSeq 2000 high-throughput sequencing platform, and each read was approximately 100 bp long to remove the reads containing adapter contamination, low-quality bases and undetermined bases. Then, the sequence quality was verified by FAST QC (http://www.bioinformatics.babraham.ac.uk/projects/fastqc/), and high-quality clean reads were obtained. Transcriptome data used in this study have been uploaded to NCBI (https://blast.ncbi.nlm.nih.gov/), and the login number is PRJNA1112305.

The clean reads were aligned to the *P. japonicus* genome using HISAT2 software. Assembly and calculation of expression values for each transcript were performed using HISAT2, StringTie, and Ballgown (Wang et al., 2022). ExpressAnalyst online software was utilized for differential expression analysis. In the process of screening differentially expressed genes, the threshold for differential gene screening was set at log2 (fold change) > 2 or log2 (fold change) < 0.05, with a significance level of P < 0.05. TBtools-ll v2.084 was used to generate volcano plots of differentially expressed genes, and enrichment analysis of GO and KEGG pathways was conducted. A differential gene heatmap was generated using https://www.bioinformatics.com.cn, an online platform for data analysis and visualization. The biosynthesis pathway of flavonoids in *P. japonicus* was determined using ChemBioDraw Ultra 12.0 software.

### 2.4 Correlation analysis of metabolic transcriptomics

WGCNA is a systems biology method used to describe gene correlation patterns between different samples (Langfelder and Horvath, 2008). It can identify sets of genes that exhibit highly coordinated changes, providing insights into the underlying biological mechanisms driving coordinated gene expression. To screen the key transcription factors involved in the biosynthesis of differential flavonoids, the local WGCNA R software package was used in this study, to analyze the weighted gene coexpression network. After the cluster observation of the data preparation samples, the soft power was set to 20 and the abline to 0.9. The weighted adjacency matrix between genes was constructed by the blockwiseModules function, and the gene coexpression network was divided into different modules (mergeCutHeight = 0.25, minModuleSize = 30). Modular characteristic genes were defined as the first principal component of a given module and were used to represent the expression profile of modular genes in each sample, and Pearson correlation analysis was performed between each modular characteristic gene and flavonoids.

### 2.5 Quantitative real-time PCR analysis

Total RNA was extracted from the samples using an EasyPure Plant RNA Kit (TransGen Biotech) supplemented with RNase-free DNase I (TransGen Biotech) to eliminate DNA contamination. The concentration and quality of the RNA samples were assessed using a NanoPhotometer N50 (Implen, GER). Subsequently, the Perfect-Start Uni RT−qPCR Kit (TransGen Biotech) was used to reverse transcribe RNA into cDNA, followed by two-step quantitative real-time PCR using a Roche Light Cycler 96 (SYBR-GREEN I; no passive reference dye). The *β-actin* gene was utilized as the internal control. The 2^−ΔΔCT^ method was used for determining the relative expression of genes. The primers used for qRT−PCR were synthesized by Sangon Biotech (Shanghai, China), and their sequences are listed in **Supplementary Table S4**.

## 3 Results

### 3.1 Metabolomic analysis of flavonoids in *P. japonicus*

**Figure 1:**
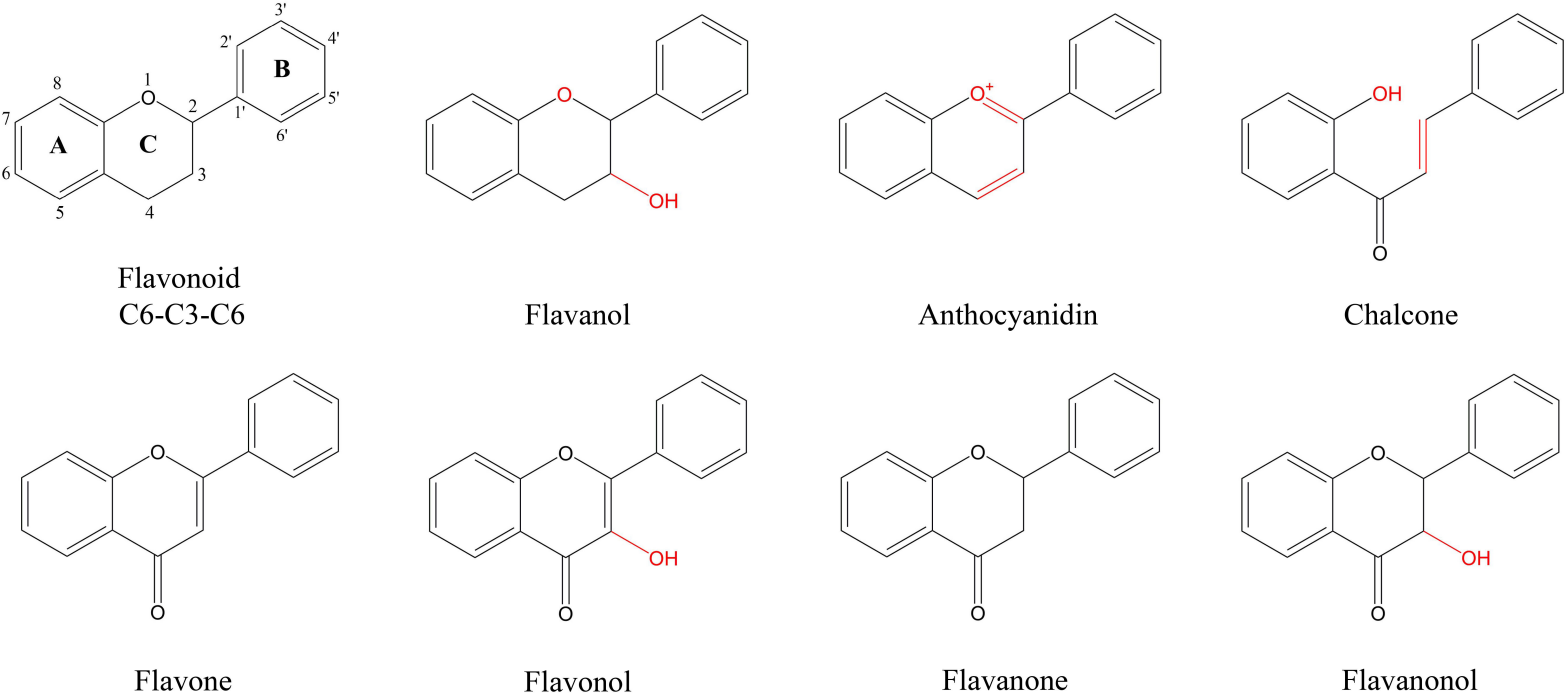
Characteristic structure of flavonoids of flavonoids metabolites in *P. japonicum*. Flavonoid is the basic structure of a C6-C3-C6. The flavonoids of *P. japonicum* were mainly classified into Flavones; Flavonols; Flavanones; Flavanonols; Flavanols; Anthocyanidins; Chalcones.

**Figure 2:**
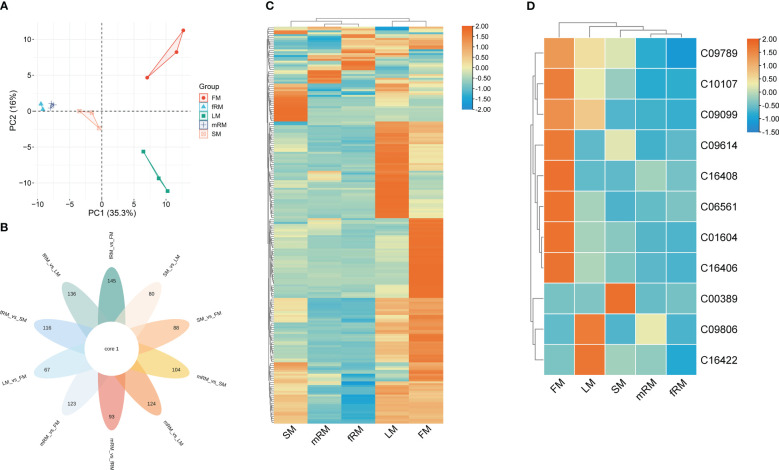
Characteristic analysis of flavonoids in different tissues of *P. japonicum*. **(A)** Factor map of the PCA performed on 15 tissue samples and 209 Flavonoid metabolites, Five cluster groups were identified corresponding to main root, fibrous root, stem, leaf and flower. **(B)** Venn diagram analysis of the number of differences between ten groups of samples for flavonoid metabolites. **(C)** Cluster analysis of DAFs. **(D)** Cluster analysis of DAFs mapped to the Ko00941 pathway. Orange shows the higher accumulation of flavonoids in different tissues of *P. japonicum*, and blue shows the lower accumulation of flavonoids.

**Figure 3:**
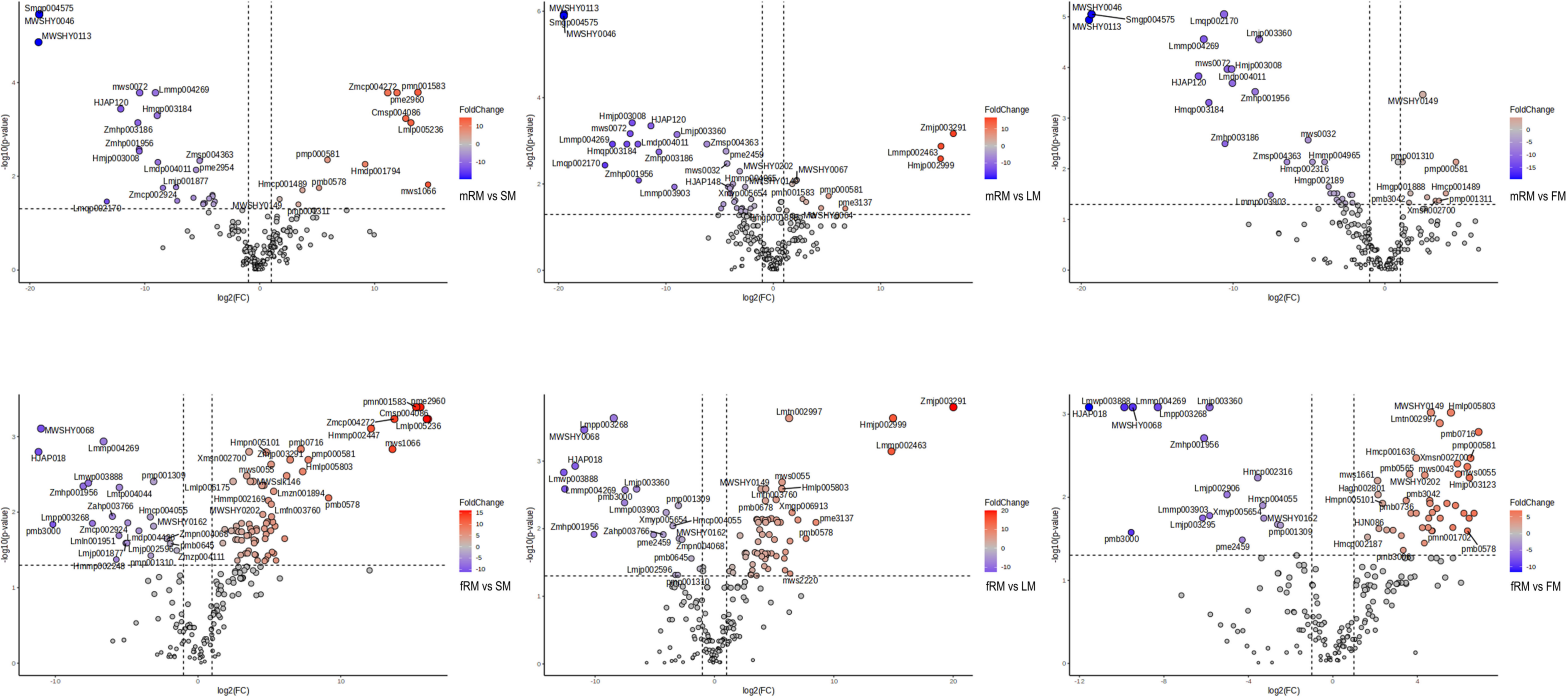
Volcano plot analysis of differential flavonoids in different tissues of *P. japonicum*. X-axis: Log2 (Fold Change). Y-axis: -Log10 (p-value). Red represents the accumulation of up-regulated flavonoids, blue is down-regulated.

UPLC-MS/MS was used to analyze the main roots, fibrous roots, stems, leaves and flowers of *P. japonicus* (the ion chromatograms in **Supplementary Figure S1**), which were named mRM, fRM, SM, LM and FM, respectively. A total of 209 flavonoids (**Supplementary Table S1**) in seven categories (**Figure 1**) were detected in five tissues, among which flavonols and flavones accounted for the largest proportion, with 106 (50.72%) and 63 (30.14%), respectively. In addition, flavanones (16), chalcones (12), flavanonols (5), flavanols (4) and anthocyanins (3) were the least abundant. PCA analysis revealed a distribution where PC1 accounted for 35.3% and PC2 accounted for 16.0%, which indicated that the five samples were separated, with great differences between groups and small differences within groups (**Figure 2A**). According to VIP ≥ 1 and fold change ≥ 2 or ≤ 0.5, 145 differentially accumulated flavonoids were identified in the fRM vs. FM group, 136 in the fRM vs. LM group, 116 in the fRM vs. SM group, 67 in the LM vs. FM group, 123 in the mRM vs. FM group, 93 in the mRM vs. fRM group, 104 in the mRM vs. SM group, 88 in the SM vs. FM group, 80 in the SM vs. LM group, and 124 in the mRM vs. LM group, with only one common flavonoid metabolite observed (**Figure 2B**). Furthermore, the differentially accumulated metabolites with P < 0.05 were analyzed via a volcano plot (a volcano plot of underground tissue vs. aboveground tissue is shown in **Figure 3**, and the other groups are shown in **Supplementary Figure S2**). The top five differentially accumulated flavonoids were screened in aboveground tissues (stems, leaves and flowers) and underground tissues (main roots and fibrous roots), among which 11 flavonoid metabolites were selected in aboveground tissues (Lmmp004269, HJAP120, MWSHY0046, Smgp004575, MWSHY0113, Lmqp002170, MWSHY0068, pmb3000, Lmwp003888, HJAP018 and Zmhp001956), and 19 differentially accumulated flavonoids were obtained in underground tissues (pmp000581, Hmcp001489, pmp001311, Xmsn002700, mws0043, Zmjp003291, Lmmp002463, Hmjp002999, pme3137, mws1066, pmn001583, Lmlp005236, Cmsp004086, pme2960, pmb0716, Hmjp003123, MWSslk146, pmb0578 and Zmcp004272).

Cluster analysis of the contents of flavonoid metabolites in different tissues revealed significant differences in the contents of flavonoids in LM and FM, and flavonoid metabolites accumulated more in LM and FM than in other tissues. At the same time, there were significant differences between aboveground and underground tissues (**Figure 2C**). Only 26 of the 209 flavonoids were annotated in the KEGG biosynthesis pathway, among which 11 flavonoids were annotated in the ko00941 (flavonoid biosynthesis) biosynthesis pathway, including two flavonoids (C10107 and C00389), five flavanones (C09806, C09614, C09099, C09789 and C16422) and four chalcones (C16408, C01604, C06561 and C16406). Two flavonoid metabolites were annotated in the biosynthesis pathway of Ko00942 (anthocyanin biosynthesis), and the other 13 flavonoid metabolites were annotated in the pathway of Ko00944 (flavone and flavonol biosynthesis). Furthermore, the contents of flavonoid metabolites annotated to Ko00941 in different tissues were clustered. The results showed that (**Figure 2D**) the accumulation of 11 flavonoids was greatest in the flowers, followed by the leaves and finally the stems, while the accumulation of flavonoids was lowest in the main roots and fibrous roots.

### 3.2 Transcriptome analysis of *P. japonicus*

**Figure 4:**
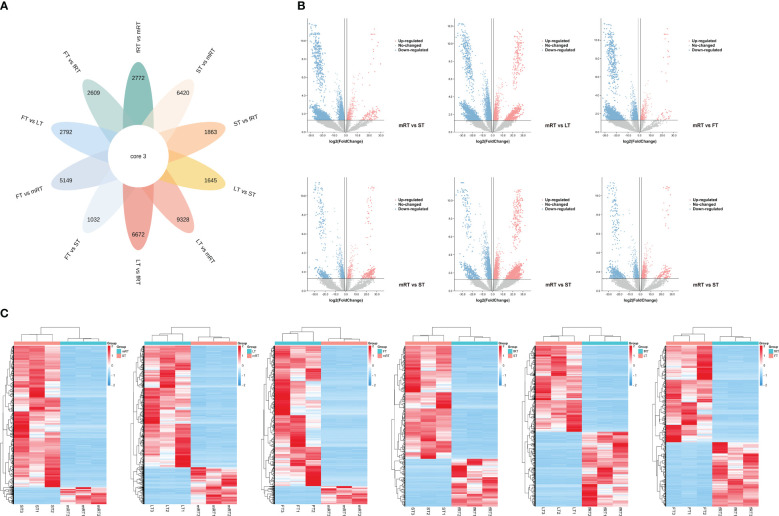
Differential transcript analysis in different tissues. **(A)** Venn diagram analysis of the number of differences between ten groups of samples for DEGs. **(B)** Volcano plot analysis of differential DEGs in different tissues of *P. japonicum*. **(C)** Heatmap analysis of differential gene expression in different tissues of *P. japonicum*. The gene expression values were presented as log2-transformed normalized TPM values. The pink is the up-regulated gene and the blue is the down-regulated gene.

The samples analyzed by metabolomics were named mRT, fRT, ST, LT and FT. There were 61,942 annotated genes, and an FDR < 0.05 and a |log2 FC| ≥ 2 were used as the criteria for screening genes for significant differences in expression. DEGs were screened from the transcriptome data, and Venn diagram (flower plot) visualization was performed (**Figure 4A**). **Figure 4B** shows that 9,328 DEGs were identified in the LT vs. mRT comparison group, with 7,147 genes upregulated. In the LT vs. fRT comparison group, 6,672 DEGs were detected, 3,177 of which were upregulated. In the FT vs. mRT comparison, 5,149 DEGs were found, 4,721 of which were upregulated. In the FT vs. fRT comparison, 2,609 DEGs were identified, 1,553 of which were upregulated. The ST vs. mRT comparison revealed 6,429 DEGs, 5,917 of which were upregulated. In the ST vs. fRT comparison, 1,863 DEGs were detected, with 1,239 genes upregulated, and only 3 genes showed common differential expression across all tissues. **Figure 4C** shows that there were more DEGs in aboveground tissues (stems, leaves and flowers) than in underground tissues (main roots and fibrous roots). The clustering results of flavonoid metabolites in different tissues showed that flavonoid metabolites accumulate more in aboveground tissues, especially in leaves and flowers, indicating that the changes in flavonoid metabolite accumulation in different tissues of *P. japonicus* may be regulated by differential gene expression.

### 3.3 Enrichment analysis of differentially expressed genes in *P. japonicus*

**Figure 5:**
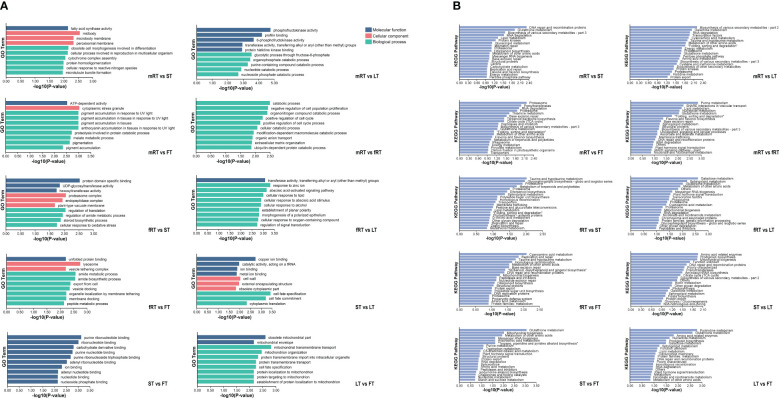
GO enrichment and KEGG enrichment of DEGs in *P. japonicum*. **(A)** GO functional enrichment analysis of DEGs in different tissues. According to the p-value ranking, the top10 were selected for data visualization analysis. **(B)** KEGG functional enrichment analysis of DEGs in different tissues. According to the P-value ranking, the top20 were selected for data visualization analysis.

The GO database was used for alignment analysis and functional annotation of DEGs. **Figure 5A** shows that the DEGs were enriched in three branches of the GO database: molecular function, cellular component and biological process. The greatest number of DEGs (454) were associated with the biological process functional classification, and most of these DEGs were associated with the biosynthesis and metabolism of biosynthetic cells. The most enriched molecular function (abundance: 116) was ATP binding, catalytic activity, and nucleic acid binding, and so on. Finally, the functional classification of cellular component (abundance: 87) was mainly enriched in membrane components, cell components and intracellular parts.

KEGG metabolic pathway annotation (**Figure 5B**) of DEGs in different tissues of *P. japonicus* revealed which metabolic pathways were closely related between different tissues and provided a basis for gene function mining in the later stages. Except for the aboveground tissues (LT vs. FV, ST vs. FT and ST vs. LT), all the other groups were annotated to Flavone and Flavonol Biosynthesis (A09100 Metabolism) or Flavone Biosynthesis (A09100 Metabolism), among which the pathway related to flavonoids annotated by DEGs between fibrous roots and aboveground tissues (FT, LT and ST) was Flavone Biosynthesis (A09100 Metabolism). Flavonoids related to the DEGs between the main roots and the fibrous roots and between the main roots and the aboveground tissues were related to flavone and flavonol biosynthesis and flavone biosynthesis. Furthermore, using the P values, the enrichment pathways of the DEGs were visually analyzed. The results showed that the 15th, 19th and 8th pathways of mRT vs. FT, mRT vs. ST and mRT vs. fRT were all involved in flavone and flavonol biosynthesis. The results showed that metabolic flux may change harmoniously with transportation in different organs of plants, and the differential accumulation of DEGs involved in the flavonoid pathway in different tissues of *P. japonicus* aboveground and underground is of potential significance.

### 3.4 Annotation and analysis of the flavonoid biosynthesis pathway

To better understand the relationship between genes and metabolites, the sequencing data were comprehensively and systematically analyzed, and the transcription and metabolite levels of *P. japonicus* and the flavonoid biosynthesis pathway were further analyzed. Thirteen kinds of DAFs were identified in different tissues of *P. japonicus*, including flavones (luteolins, chrysoeriols, apigenins and tricins), flavonols (quercetins, isorhamnetins and kaempferols), flavanones (hesperetins, eriodictyols and naringenins), flavanonols (dihydrokaempferols) and anthocyanidins (cyanidins and pelargonidins). Furthermore, 13 kinds of key enzymes genes (including the genes encoding key enzymes involved in the phenylpropanoid biosynthesis pathway) were identified among the DEGs according to the annotation results of the EC number and KEGG pathway (Ko00940, Ko00941, Ko00942, Ko00943 and Ko00944). These genes included 4-coumarate-CoA ligase (Pj4CL1-Pj4CL4), anthocyanidin synthase (PjANS1-PjANS3), trans-cinnamate 4-monooxygenase (PjC4H1-PjC4H3), chalcone isomerase (PjCHI1-PjCHI13), chalcone synthase (PjCHS1-PjCHS7), cytochrome P450 monooxygenase (PjCYP1-PjCYP14), dihydroflavonol 4-reductase (PjDFR1-PjDFR4), naringenin 3-dioxygenase (PjF3H1-PjF3H3), flavonoid 3’-monooxygenase (PjF3’H1-PjF3’H3), flavonol synthase (PjFLS1-PjFLS2), phenylalanine ammonia-lyase (PjPAL1-PjPAL4) and glycosyltransferase (PjUGT1-PjUGT6). All annotated genes in this pathway had multiple homologous genes. These results indicate that there are complex biosynthesis pathways for flavonoid metabolites in *P. japonicus*.

**Figure 6:**
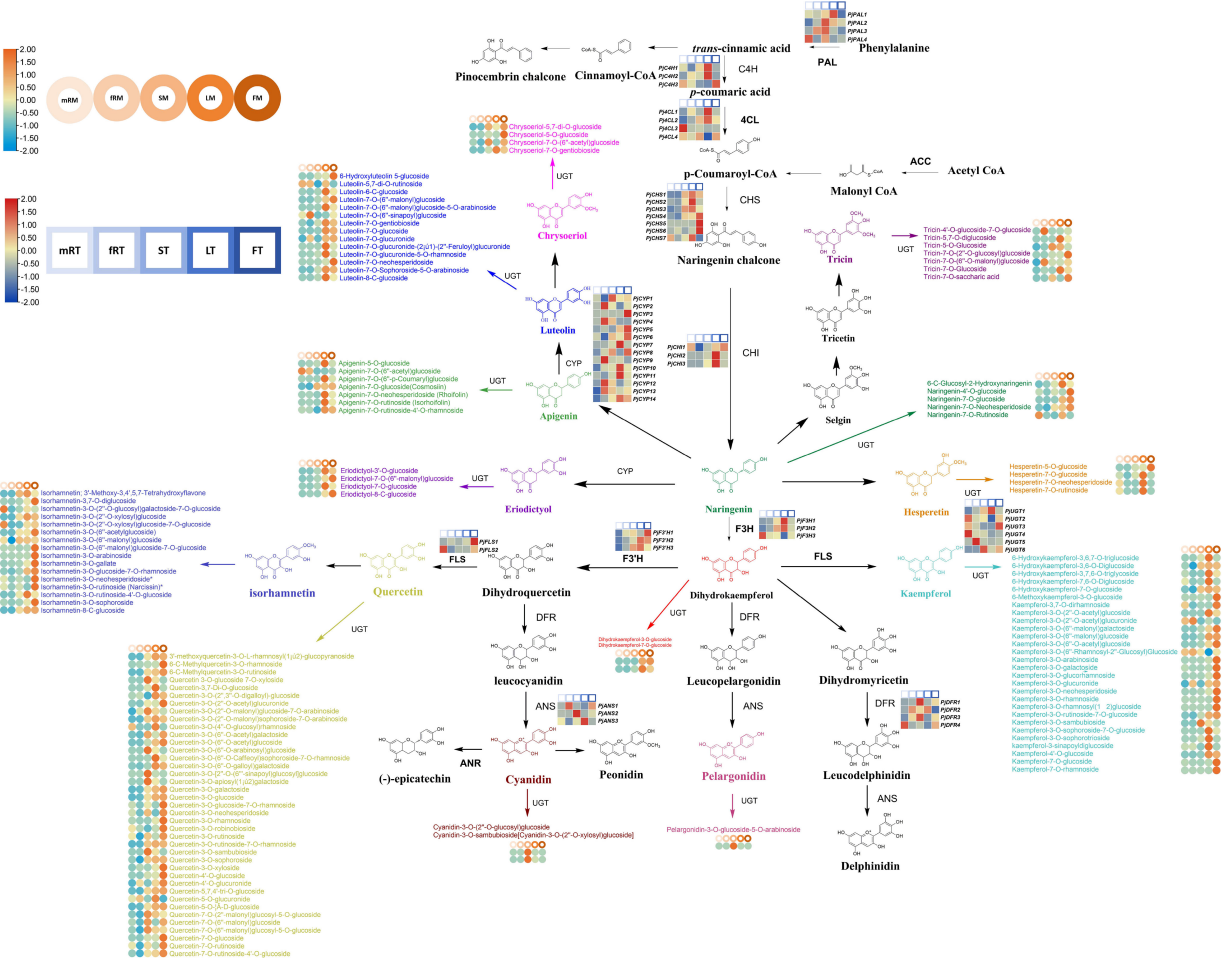
Flavonoid biosynthetic pathway of *P. japonicum*. The square heatmap represents the expression levels of differentially expressed key enzyme genes in different tissues, and TPM was used for expression data. Circles heatmap represent the accumulation of differential flavonoid metabolites in different tissues.

Based on the reported plant flavonoid metabolism pathway, the metabonomics and transcriptomics datasets of *P. japonicus* were integrated, and a heatmap was constructed according to the expression of candidate genes involved in the flavonoid metabolism pathway to evaluate the tissue-specific expression pattern of the flavonoid biosynthesis pathway in *P. japonicus* and to preliminarily assess the flavonoid biosynthesis pathway in *P. japonicus* (**Figure 6**; **Supplementary Table S2**). The results showed that the candidate genes were differentially expressed in five tissues, and more candidate genes of the flavonoid pathway were in LT and FT, followed by mRT and fRT. An in-depth exploration of the characteristics of candidate genes revealed that *Pj4CL*, *PjC4H*, *PjCHI*, *PjF3H* and *PjF3’H* had relatively high expression levels in LT, followed by FT, ST and mRT, such as *Pj4CL1*, *Pj4CL2*, *PjC4H1*, *PjC4H2*, *PjCHI2*, *PjCHI3*, *PjF3H1*, *PjF3H2* and *PjF3H3*. The genes with relatively high expression levels in the FT were *PjFLS*, *PjCYP*, *PjCHS* and *PjCHR*, such as *PjCHS4*, *PjCHS5*, *PjCHS6*, *PjCYP3*, *PjCYP5*, *PjCYP6*, *PjCYP8* and *PjFLS1*. In ST, *PjANS*, *PjDFR* and *PjPAL*, including *PjANS2*, *PjDFR1*, *PjDFR3*, *PjPAL2* and *PjPAL3*, had relatively higher expression levels. However, six *PjUGT* were highly expressed in all five tissues. The accumulation of flavonoid metabolites was analyzed via a heatmap. The results showed that luteolins mainly accumulated in leaves, followed by flowers, and that a small amount of luteolins accumulated in the main roots and fibrous roots. Chrysoeriols mainly accumulated in flowers, followed by stems and leaves; apigenins mainly accumulated in leaves, and the accumulation in the other four tissues was relatively low; tricins were mainly distributed in leaves, flowers and fibrous roots, followed by stems. Quercetins, isorhamnetins and kaempferol (flavonols) were mainly distributed in flowers, followed by leaves and stems. Hesperetins, eriodictyols and naringenins (flavanones) mainly accumulated in flowers and leaves, and naringenin also accumulated in the main roots. Dihydrokaempferols mainly accumulated in leaves and flowers, while cyanidins and pelargonidins (anthocyanidins) only accumulated in stems. The differential accumulation of these flavonoids may mainly be the result of differential expression of the key enzyme genes.

Comprehensive analysis of the results revealed that in the pathway from trans-cinnamic acid to flavanonol, flavones (luteolins, chrysoeriols, apigenins and tricins) mainly accumulated in leaves or flowers, and the contents of flavanones (hesperetins, eriodictyols and naringenins), flavonols (quercetins, isorhamnetins and kaempferols) and flavanonols (dihydrokaempferols) exhibited greater accumulation in both flowers and leaves. This part of the biosynthetic pathway was mainly regulated by *PjC4H*, *Pj4CL*, *PjCHS*, *PjCHI*, *PjF3H*, *PjF3’H*, *PjCYP*, and *PjPAL*, with these key enzyme-encoding genes predominantly expressed at high levels in leaves and flowers. The increased accumulation of pelargonidins and cyanidins in stems was mainly regulated by *PjDFR* and *PjANS*, and these two key enzyme-encoding genes were highly expressed mainly in the stems. Furthermore, *PjUGT* exhibited high expression levels in various tissues, such as the highly expressed *PjUGT1* in LT, *PjUGT3* in FT, *PjUGT6* in ST, *PjUGT5* in fRT, and *PjUGT2* and *PjUGT4* in mRT. This observation indicates that the formation of various flavonoid metabolite glycosides in different tissues is widely regulated by PjUGT in *P. japonicus*. Further qRT−PCR analysis was performed on the candidate pathway genes. The results showed that the expression patterns of 12 candidate genes (*Pj4CL1*, *PjPAL1*, *PjANS1*, *PjC4H1*, *PjCHI1*, *PjCHS1*, *PjCYP1*, *PjDFR1*, *PjF3H1*, *PjF3’H1*, *PjFLS1 and PjUGT1*) were largely consistent with the RNA-seq data (**Supplementary Figure S3**).

### 3.5 Coexpression network construction and key module screening

**Figure 7:**
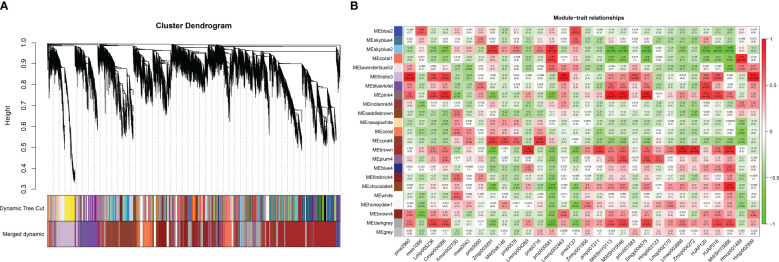
WGCNA analysis. **(A)** Hierarchical clustering dendrogram of identified co-expressed genes in modules. The same color indicates that the corresponding gene on the clustering tree belongs to the same module. **(B)** The correlation coefficient between the module and 30 differential flavonoid metabolites is described by the color of each cell at the row-column intersection. The Pearson correlation coefficient was used to plot the eigenvalues of the module and the physiological data. Red represents a positive correlation, and green represents a negative correlation. The color scale on the right shows module-trait correlation from -1 to 1.

The relationships between the expression patterns of flavonoids that differentially accumulated in 11 aboveground tissues (stems, leaves and flowers) and 19 underground tissues and the gene regulatory networks related to their accumulation were screened by WGCNA. The results showed that the genes obtained by transcriptome sequencing were mainly divided into 23 modules (**Figure 7**). Further analysis focused on modules with r > 0.8, revealing that six modules correlated with flavonoid metabolites. Among them, three modules showed a correlation of r > 0.8 with only one flavonoid metabolite: skyblue2 with pmp000581 (r = 0.81), coral1 with hmcp001489 (0.81), and chocolate4 with MWSHY0068 (0.85). Additionally, three modules exhibited r > 0.8 correlation with multiple flavonoid metabolites: thistle3 with pme2960 (r = 0.91), lmmp002463 (0.95), and hmjp002999 (0.92); pink4 with msp004086 (r = 0.82), MWSHY0113 (0.93), MWSHY0046 (0.94), and smgp004575 (0.94); and brown with lmmp004269 (r = 0.84), lmwp003888 (0.88), zmcp004272 (0.88), and 0.88MWSHY0068 (0.81). Among these 14 flavonoid metabolites, seven exhibited increased accumulation in aboveground tissues (MWSHY0113, MWSHY0046, smgp004575, lmmp004269, lmwp003888, MWSHY0068, and MWSHY0068), while seven exhibited increased accumulation in underground tissues (pmp000581, hmcp001489, pme2960, lmmp002463, hmjp002999, cmsp004086, and zmcp004272).

Based on the results of the metabonomics analysis, extensive and representative experimental groups (main roots vs. flowers and fibrous roots vs. leaves) were selected for in-depth exploration. Using the transcript annotation information, transcription factors (TFs) within the modules were selected (**Supplementary Table S3**). In the coral1 module, annotations included 1 bHLH, 1 WRKY, 1 WD40, and 1 AP2. The chocolate4 module was annotated with 1 bZIP, 1 bHLH, 1 WRKY, and 2 WD40 genes. The thistle3 module was annotated with 3 bZIPs, 4 bHLHs, 10 MYBs, 3 WRKYs, 7 WD40s, and 6 AP2s. The pink4 module was annotated with 1 bZIP, 4 MYB, 1 WRKY, and 1 GRAS. The brown module was annotated with 5 bZIPs, 13 bHLHs, 34 MYBs, 4 WRKYs, 21 WD40s, 4 GRASs, and 23 AP2s. WRKYs were annotated in 5 modules, followed by bZIPs, bHLHs, and WD40s, which were each annotated in 4 modules. Further analysis of the expression levels of TFs in modules related to the significant accumulation of flavonoid metabolites in the aboveground parts of *P. japonicus* revealed that 116 relevant TFs were predominantly highly expressed in leaves, and a few were highly expressed in flowers or stems. No specific expression of relevant TFs was found in the main roots or fibrous roots (**Supplementary Figure S4**).

## 4 Discussion

Flavonoids have a variety of biological activities, and their biosynthesis is initiated via the phenylpropanoid metabolism pathway. As one of the secondary metabolites of plants, they play a crucial role in plant growth and development processes (Winkel-Shirley, 2001). Flavonoids are related to the transport of plant growth regulators, affect the levels of hormones such as auxin and cytokinin, and play an important role in regulating root growth and nutrient absorption (Kuhn et al., 2017; Bhakta et al., 2022). Flavonoids can be used as phytochemical defense substances, induce and participate in the defense response of tissues and pathogenic microorganisms, and have antiviral and antibacterial activities (Ramaroson et al., 2022; Zhao et al., 2024). In this study, a total of 209 flavonoid metabolites were detected by metabolomics, among which 133 metabolites were annotated to the flavonoid biosynthesis pathway. The majority of flavonoid metabolites accumulate significantly in aboveground parts of the plant, particularly in flowers and leaves, followed by stems (mostly anthocyanidins), but less so in underground parts (mainly roots and fibrous roots). Previous investigations on the contents of phenolics, flavonoids and vitamins in different parts of Korean ginseng (*P. ginseng*) showed that the contents of total phenolics and flavonoids in leaves were the highest, followed by those in the main roots and root hairs, and catechin and kaempferol were the main flavonoids in leaves (Kim, 2016). The high accumulation of flavonoid metabolites in the flowers of *P. japonicus* may give flowers a specific smell, attract pollinators such as insects, and promote pollen spread and reproduction (Xue et al., 2023). Moreover, flavonoids in leaves may be related to photosynthesis and participate in regulating photosynthetic efficiency and plant adaptation to light (Zhang et al., 2023a). Generally, the specific expression of flavonoid metabolites in different tissues of *P. japonicus* may reflect the differences and characteristics of functional differentiation, environmental adaptation and signal transduction of *P. japonicus*.

For the same genus of *P. japonicus*, such as *P. ginseng*, *P. notoginseng* and *Panax quinquefolius*, researchers have explored the biosynthesis and accumulation patterns of flavonoids and conducted systematic and in-depth research on their underground parts as well as their aboveground parts (Xiao, 2010). In this study, the transcriptomes of different tissues of *P. japonicus* were sequenced and analyzed, and 13 kinds of key enzyme-encoding genes of the flavonoid biosynthesis pathway were identified, among which the genes with the highest expression in flowers were *PjC4H3*, *Pj4CL4*, *PjCHS4*, *PjCHS5*, *PjCHS6*, *PjCHI1*, *PjF3’H1*, *PjFLS1*, *PjCYP3*, *PjCYP5*, *PjCYP6*, *PjCYP8*, and *PjUGT3*. Genes with higher expression in leaves included *PjPAL1*, *PjC4H1*, *PjC4H2*, *Pj4CL1*, *Pj4CL2*, *PjCHS1*, *PjCHS2*, *PjCHS3*, *PjCHS7*, *PjCHI2*, *PjCHI3*, *PjF3H1*, *PjF3H2*, *PjF3’H2*, *PjF3’H3*, *PjDFR2*, *PjANS3*, *PjCYP7*, *PjCYP10*, *PjCYP11*, *PjCYP14*, and *PjUGT1*. In a study on the low nitrogen-driven biosynthesis of saponins and flavonoids in *P. notoginseng*, it was found that nitrogen deficiency promoted the accumulation of amino acids and sugars, thus providing precursor metabolites for the biosynthesis of flavonoids and triterpenoid saponins. The downregulation of key structural genes involved in the flavonoid biosynthesis pathway, especially F3H, may lead to a decrease in flavonoid content (Cun et al., 2024). Research on the biosynthesis of flavonoids such as anthocyanins in the purplish-green aerial stems of *P. notoginseng* revealed that structural genes (SGs) and transcription factor genes (TFs) were mainly CHI_1_ and ANS, bHLH_3_, MYB_1_ and WD40_3_, and bHLH_1_ played a negative regulatory role (Zhang et al., 2022b). In summary, the flavonoid biosynthesis pathway is directly related to the biosynthesis and accumulation of flavonoids (Davies et al., 2020).

PAL is the first rate-limiting enzyme in the phenylpropanoid metabolic pathway, controlling the conversion of primary metabolites to secondary metabolites. The enzyme 4CL converts p-coumaric acid into p-coumaroyl-CoA. The enzyme is also involved in controlling the efflux of p-coumaroyl-CoA from different branches of the phenylpropanoid pathway and in its conversion to caffeic acid and ferulic acid in lignin biosynthesis (Li et al., 2015). Therefore, *PjPAL* and *Pj4CL*, which have the same expression patterns as other structural genes involved in flavonoid biosynthesis, may actively participate in the regulation of flavonoid metabolite biosynthesis, while PjPAL and Pj4CL, which have slightly different patterns, may play a role in lignin biosynthesis. The CHS gene of *A. thaliana* regulates flavonoid accumulation and abiotic stress tolerance (Wang et al., 2018). The expression of CtCHI-N in safflower is related to the biosynthesis of flavonoids at the flowering stage (Ren et al., 2019). Therefore, the specific expression of *PjCHS* and *PjCHI* in flowers and leaves may also be one of the main reasons for the increased accumulation of flavonoid metabolites in the aboveground tissues of *P. japonicus*. DFR is the key enzyme of the anthocyanidin biosynthesis pathway. Research on *Rubus chingii* revealed that the competition between FLS and DFR regulated the metabolic flux distribution of flavonoids and PAs (proanthocyanidins) (Lei et al., 2023). *PjDFR1* and *PjDFR3*, which may promote the accumulation of anthocyanidin metabolites in *P. japonicus*, are highly expressed in the stems of *P. japonicus*. ANS, FLS and F3H are members of the 2-oxoglutarate-dependent dioxygenase (2-ODD) superfamily in plants and play important roles in flavonoid biosynthesis (Saxena et al., 2023). When the ANS gene is repressed, more carbon flows to flavonol biosynthesis, while when the FLS gene is repressed, more anthocyanidins accumulate (Wang et al., 2019). F3H determines the contents of anthocyanins and flavonols in plants (Dai et al., 2022). In this study, *PjANS2* was specifically expressed in the stems, while *PjFLS1* and *PjFLS2* were expressed at low levels in the stems, which may also be the reason for the high anthocyanidin content in the stems of *P. japonicus*. In addition, the proportion of flavonol metabolites measured reached 50.72%, and the relatively high expression of *PjF3H* in flowers and main roots may be the key to the accumulation of flavonol metabolites in *P. japonicus*. Based on the above results, the specific expression of flavonoid biosynthesis pathway genes helps reveal the regulatory mechanism, metabolic regulation and environmental adaptation of the flavonoid biosynthesis pathway in *P. japonicus*.

The glycosylation of flavonoids in plants is mostly catalyzed by UDP-glycosyltransferase (UGT), which transfers glycosyl groups from activated sugar donors to acceptor molecules, thus generating flavonoid glycosides with diverse structures (Lyu et al., 2020; Wang et al., 2024). According to a study of the tissue transcriptome of *Ginkgo biloba*, the recombinant UGT716A1 protein has activity on a wide range of flavonoid/phenylpropanoid substrates, and its expression level paralleled the flavonoid distribution pattern in *G. biloba*, and heterologous overexpression of UGT716A1 in *A. thaliana* led to increased accumulation of several flavonol glucosides (Su et al., 2017). In addition, previous studies have shown that plant glycosyltransferases play important roles in plant growth and interactions with the environment. The heterologous overexpression of UGT76E11 has been shown to result in a significant increase in flavonoid content in transgenic *A. thaliana*, and transgenic *A. thaliana* exhibited greatly improved tolerance to salinity and drought. Moreover, the overexpression of UGT76E11 also enhanced the ability to scavenge reactive oxygen species (ROS) and increased the expression levels of many stress-related genes (Li et al., 2018). *Arachis hypogaea* AhUGT75A (UGT73CG33) is located in mitochondria and was characterized as a flavonoid 7-O-UGT by *in vitro* enzyme assays. The heterologous overexpression of AhUGT75A led to a decrease in malondialdehyde (MDA) and superoxide accumulation in *A. thaliana* and enhanced tolerance to drought and salt stress (Ouyang et al., 2023). According to the transcriptome data of this study, a total of six UGTs were initially annotated in the flavonoid metabolic pathway, and these six *PjUGT*s were relatively highly expressed in five tissues of *P. japonicus*. The *PjUGT*s wide expression in *P. japonicus* indicated that it was of great significance to the secondary metabolism, growth and development, environmental adaptation and metabolic regulation of *P. japonicus*.

The TFs reported to be involved in regulating the flavonoid metabolic pathway include MYB, bZIP, bHLH, WRKY, AP2/ERF, and WD40. These factors can directly bind to the promoters of flavonoid biosynthetic genes, thereby regulating flavonoid biosynthesis (Yan et al., 2021; Cao et al., 2024). Three R2R3-MYBs may be involved in regulating the biosynthesis of flavonoids in *P. quinquefolius* (Song et al., 2024). Coexpression analysis of the transcriptome and metabolomics data revealed that MYB, bHLH and bZIP TFs in *Torreya grandis* may be involved in regulating flavonoid biosynthesis (Zhang et al., 2022a). In this study, the TFs related to the biosynthesis of flavonoids in aboveground or underground parts of the plant were identified via WGCNA, such as bHLH, MYB, WRKY, WD40, bZIP, bHLH, GRAS and AP2, were annotated in five candidate modules, among which WRKY was widely found in five candidate modules. These key regulatory factors may actively regulate the biosynthesis of flavonoids in *P. japonicus* and promote the accumulation of flavonoid metabolites in different tissues. The significant upregulation of TFs in leaves, flowers, and stems may contribute to the increased expression of key enzyme-encoding genes involved in the flavonoid biosynthetic pathway in the aboveground organs of *P. japonicus*, thereby enhancing the accumulation of flavonoid metabolites. Flavonoids have essential physiological functions in plants, including antioxidation, antipathogen, and stress resistance functions, aiding plants in responding to external pressure and environmental changes. WRKYs play a crucial role in regulating the synthesis of various flavonoids in response to a range of abiotic stresses, such as UV-B, O3, and mechanical damage. When the transgenic calli of apple plants are exposed to UV-B radiation, MdWRKY72 directly controls anthocyanin biosynthesis by promoting MdMYB1 or indirectly regulates anthocyanin biosynthesis by interacting with MdMYB16 (Hu et al., 2020; Mao et al., 2021). In addition, McWRKY71 directly binds to McANR, thus regulating PA biosynthesis related to O3 stress in begonia (Zhang et al., 2022c, 2023b). The expression of bHLH, C2H2, ERF, bZIP, GRAS and MYB TFs in *Cunninghamia lanceolata* roots changes significantly under aluminum (Al) stress. These differentially expressed TFs may interact synergistically with flavonoid and phenylpropanoid pathway genes and play a key role in the response of *C. lanceolata* roots to aluminum toxicity (Ma and Lin, 2019). In this study, a wide variety of transcription factor families were identified in *P. japonicus*. TFs regulate the biosynthetic pathways of flavonoids, influencing their biosynthesis and improving the response of *P. japonicus* to various biotic and abiotic stresses, stress resistance and environmental adaptability, which provides crucial support for the growth and survival of *P. japonicus*.

## 5 Conclusion

In this study, both metabonomics and transcriptomics were used to elucidate the relationships between key genes involved in the flavonoid biosynthesis pathway and metabolites. The findings revealed a distinct tissue-specific accumulation of flavonoids in *P. japonicus*, with a predominant accumulation in flowers and leaves. The accumulation pattern was predominantly influenced by a variety of structural genes (PjC4H, Pj4CL, PjCHS, PjCHI, PjF3H, PjF3’H, PjCYP, PjPAL, and PjUGT) and transcription factors (PjbHLH, PjMYB, PjWRKY, PjWD40, PjbZIP, PjbHLH, PjGRAS, and PjAP2). These identified candidate genes offer valuable insights and references for further investigations into the regulation of the flavonoid biosynthesis pathway and flavonoid accumulation in *P. japonicus*. Nonetheless, the specific mechanisms underlying its biosynthesis warrant further exploration.

## Data availability statement

The datasets presented in this study can be found in online repositories. The names of the repository/repositories and accession number(s) can be found in the article/**Supplementary Material**.

## Author contributions

CC: Conceptualization, Data curation, Writing – original draft. PW: Conceptualization, Data curation, Methodology, Writing – original draft. YY: Visualization, Writing – review & editing. ZJ: Investigation, Writing – review & editing. SX: Visualization, Writing – review & editing. YL: Investigation, Methodology, Supervision, Writing – review & editing. PD: Conceptualization, Investigation, Software, Writing – review & editing.
