# Supplementary material for: Integrated metabolome and transcriptome analysis provide insight into the biosynthesis of flavonoids in Panax japonicus
Source: Front Plant Sci. 2024 Jul 29;15:1432563. doi: 10.3389/fpls.2024.1432563 (PMC11317393; doi:10.3389/fpls.2024.1432563)
Supplement: Supplementary file 2 [file DataSheet_1.docx]

Supplementary Material

| A | 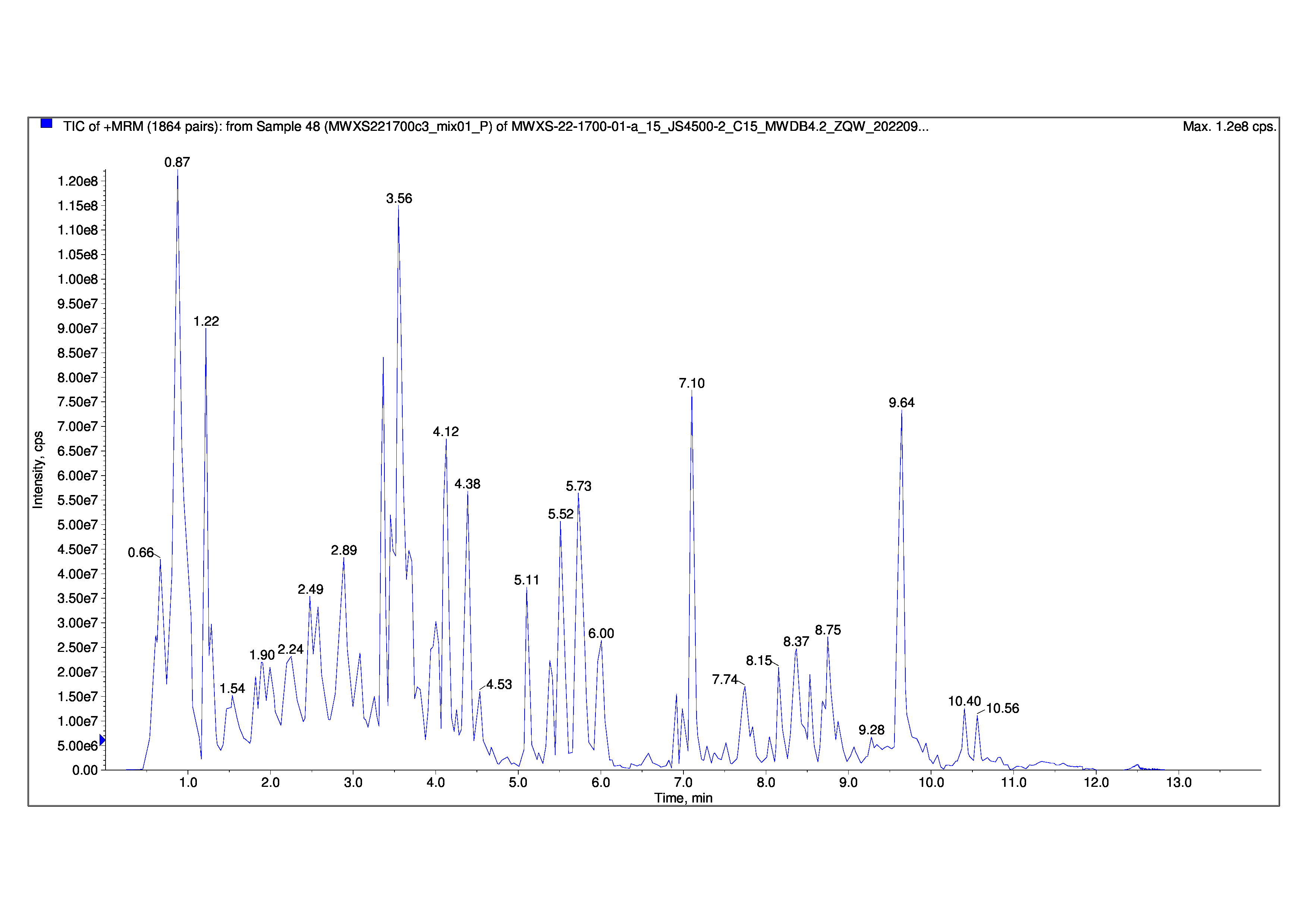 |
| --- | --- |
| B | 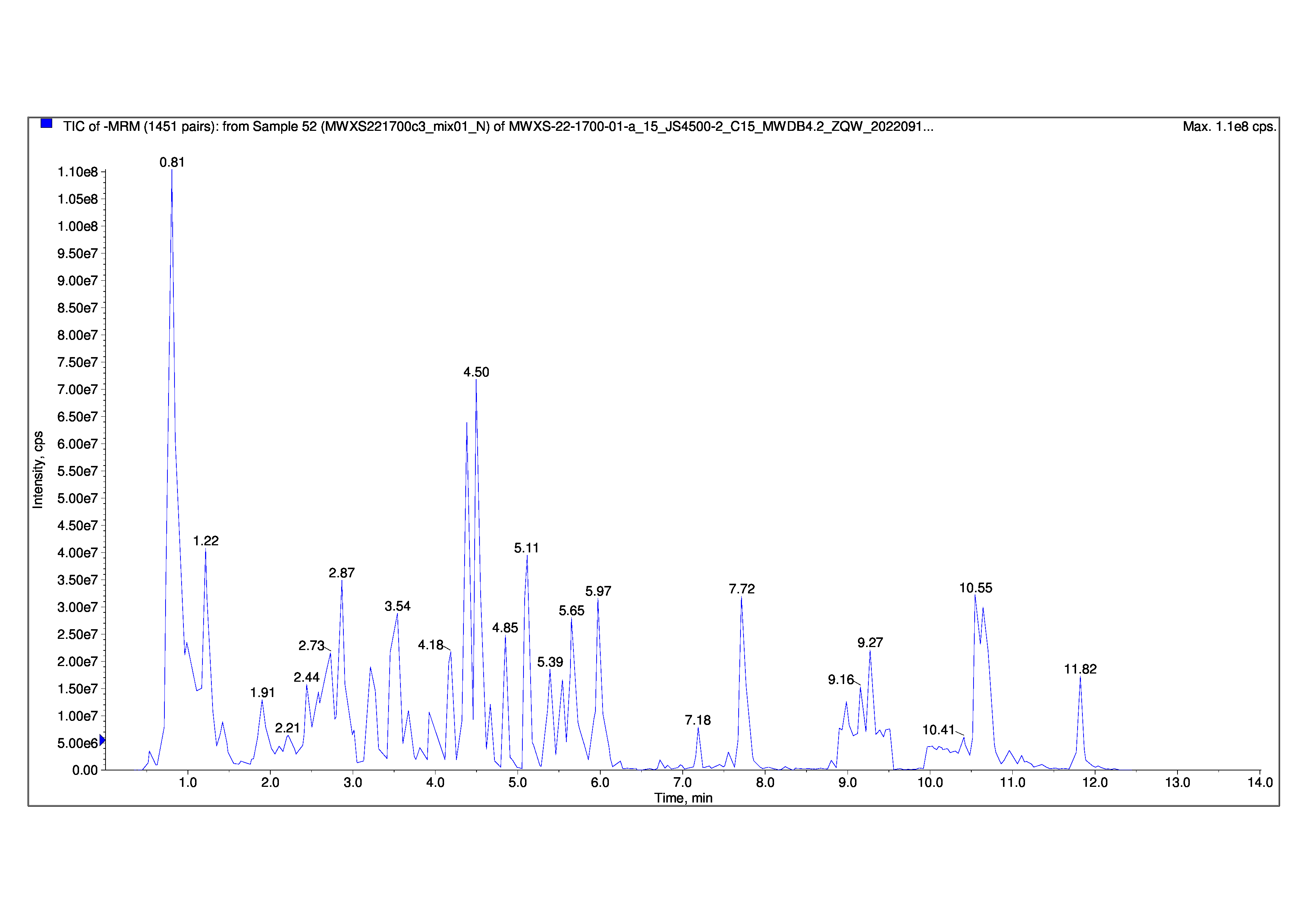 |
| Supplementary Figure S1. The software Analyst 1.6.3 was used to process the mass spectrometry data. Figures shows the total ion current diagram of mixed quality control QC samples and the multi-peak diagram of MRM metabolite detection, with the retention time of metabolite detection on the abscissa and the ion current intensity of ion detection on the ordinate. (A) Positive ion chromatography based on UPLC-MS/MS; (B) Negative chromatography based on UPLC-MS/MS. | |

| 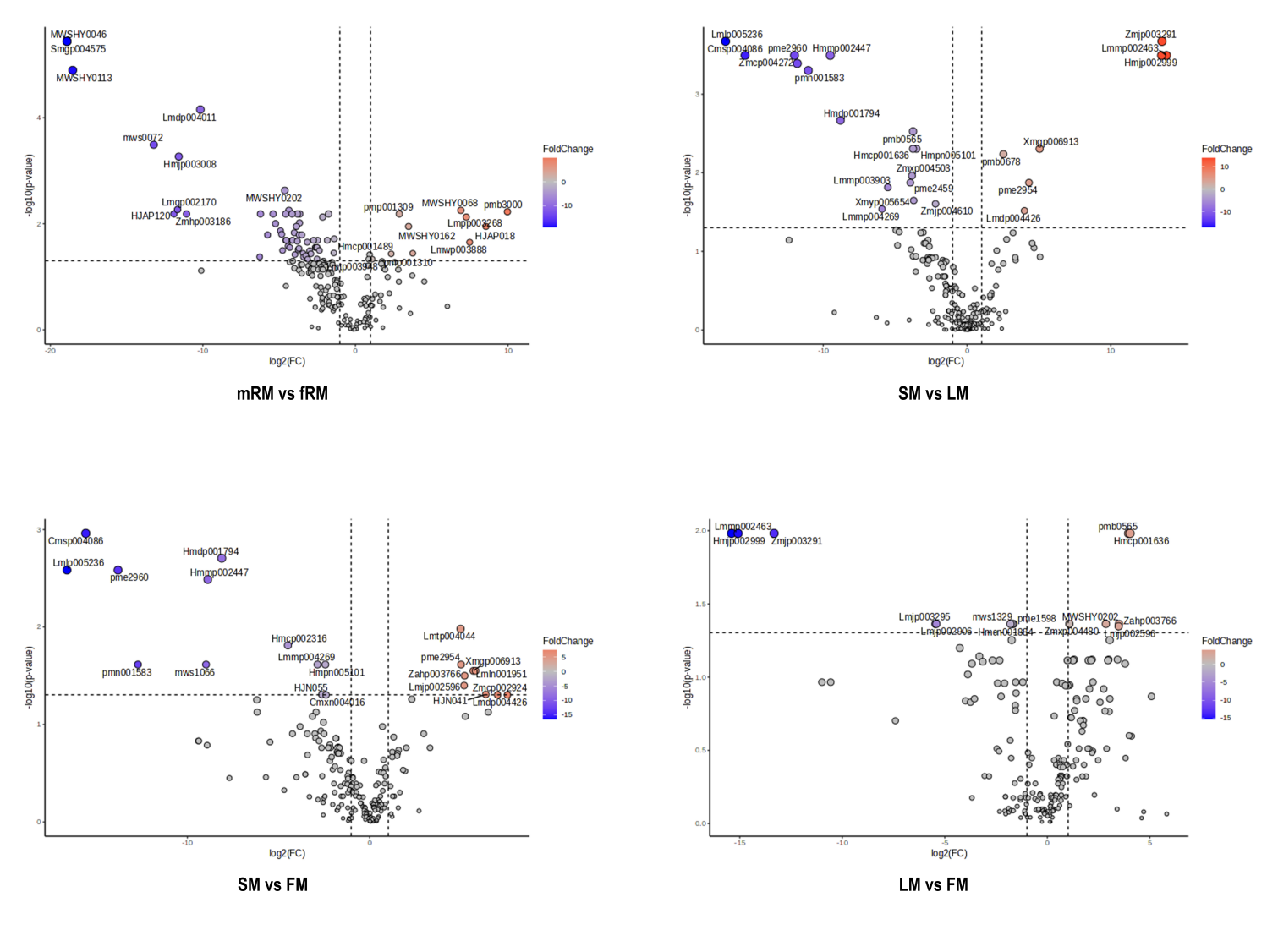 |
| --- |
| **Supplementary Figure S2.** Volcano plot analysis of differential flavonoids in different tissues of *P. japonicus*. X-axis: Log2 (Fold Change). Y-axis: -Log10 (P-value). Red represents the accumulation of up-regulated flavonoids, blue is down-regulated. |
| 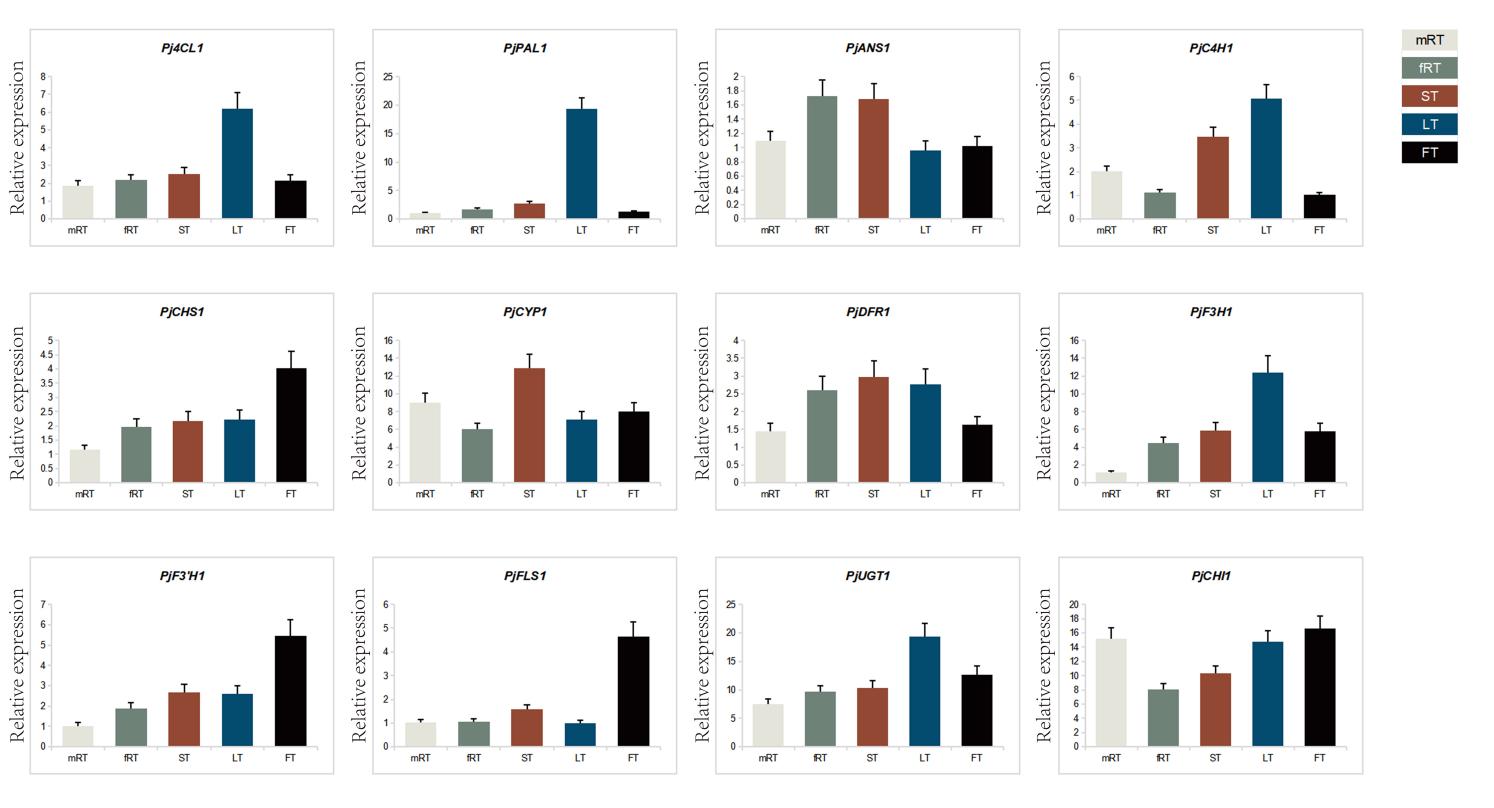 |
| **Supplementary Figure S3.** The expression of 12 candidate genes in five *P. japonicus* tissues was analyzed by qRT-PCR. Data were normalized to *β-actin* gene and vertical bars indicated standard deviation. |

| 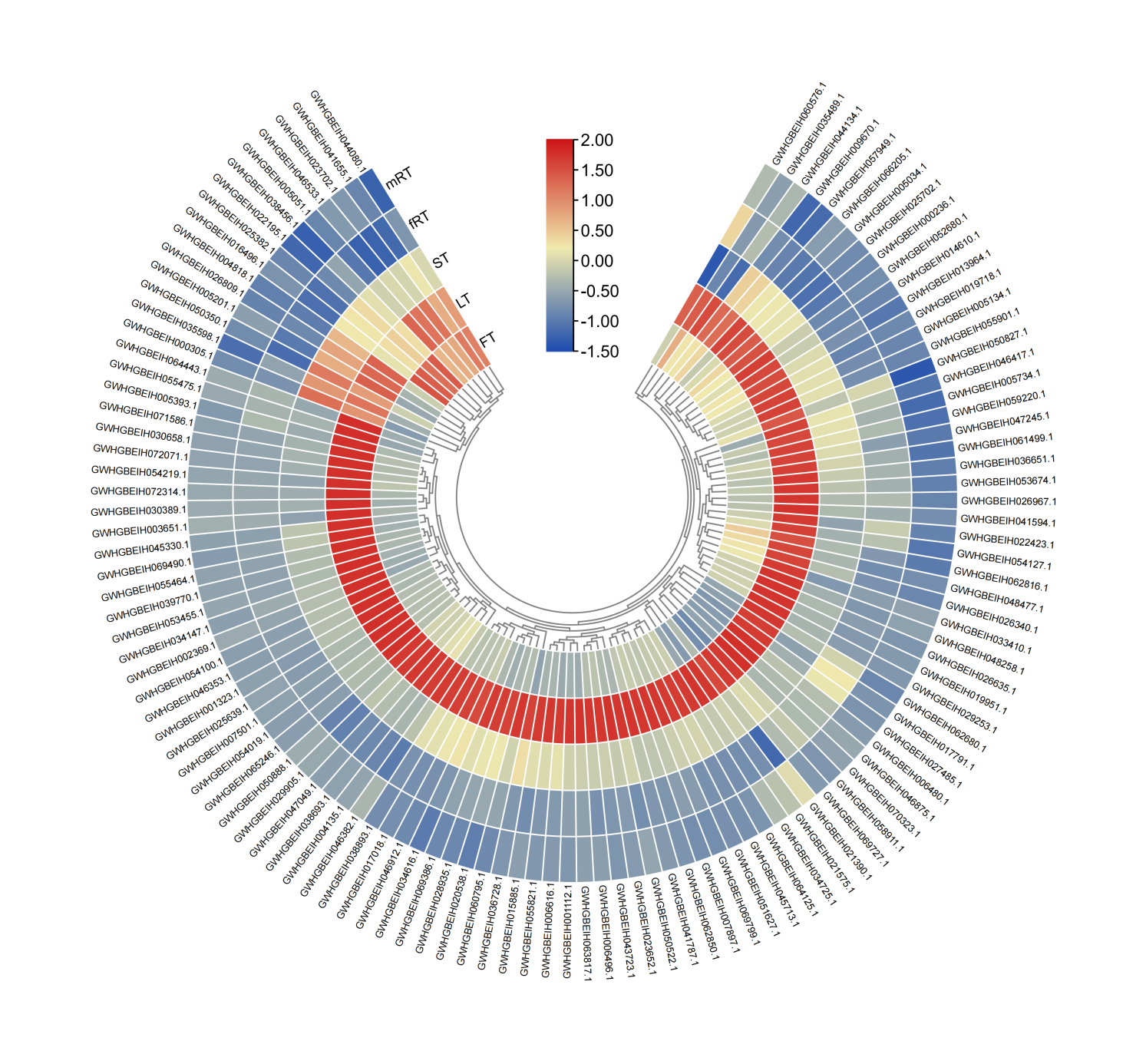 |
| --- |
| **Supplementary Figure S4.** Expression profiles of candidate transcription factors in five tissues. The gene expression values present as log2-transformed normalized TPM values. Red represents high expression, and blue represents low expression. |
